# The Competition between 4-Nitrophenol Reduction and BH_4_^−^ Hydrolysis on Metal Nanoparticle Catalysts

**DOI:** 10.3390/molecules28186530

**Published:** 2023-09-08

**Authors:** Shalaka Varshney, Dan Meyerstein, Ronen Bar-Ziv, Tomer Zidki

**Affiliations:** 1 Chemical Sciences Department, The Centre for Radical Reactions and Material Research, Ariel University, Kyriat Hamada 3, Ariel 40700, Israel; shalaka.varshney15@gmail.com (S.V.); danm@ariel.ac.il (D.M.); 2 Department of Chemistry, Ben-Gurion University of the Negev, Beer-Sheva 84105, Israel; 3 Department of Chemistry, Nuclear Research Centre Negev, Beer-Sheva 84190, Israel; bronen@post.bgu.ac.il

**Keywords:** 4-aminophenol, bimetallic nanoparticles, borohydride, borate, catalysis, competitive reactions, hydrogen evolution reaction, 4-nitrophenol reduction

## Abstract

Assessing competitive environmental catalytic reduction processes via NaBH_4_ is essential, as BH_4_^−^ is both an energy carrier (as H_2_) and a reducing agent. A comprehensive catalytic study of the competition between the borohydride hydrolysis reaction (BHR, releasing H_2_) and 4-nitrophenol reduction via BH_4_^−^ on M^0^- and M/M′ (alloy)-nanoparticle catalysts is reported. The results reveal an inverse correlation between the catalytic efficiency for BH_4_^−^ hydrolysis and 4-nitrophenol reduction, indicating that catalysts performing well in one process exhibit lower activity in the other. Plausible catalytic mechanisms are discussed, focusing on the impact of reaction products such as 4-aminophenol and borate on the rate and yield of BH_4_^−^ hydrolysis. The investigated catalysts were Ag^0^, Au^0^, Pt^0^, and Ag/Pt-alloy nanoparticles synthesized without any added stabilizer. Notably, the observed rate constants for the 4-nitrophenol reduction on Ag^0^, Ag-Pt (9:1), and Au^0^ are significantly higher than the corresponding rate constants for BH_4_^−^ hydrolysis, suggesting that most reductions do not proceed through surface-adsorbed hydrogen atoms, as observed for Pt^0^ nanoparticles. This research emphasizes the conflicting nature of BH_4_^−^ hydrolysis and reduction processes, provides insights for designing improved catalysts for competitive reactions, and sheds light on the catalyst properties required for each specific process.

## 1. Introduction

Borohydride, BH_4_^−^, is a strong reducing agent [1,2,3], commonly used as a reducing agent or as a source of energy as H_2_ formed via solvolysis in protic solvents; Reaction (1).

BH_4_^−^ + 4ROH → 4H_2_ + B(OR)_4_^−^ (1)

Also, NaBH_4_ is an excellent energy carrier due to its (i) low weight and volume; (ii) high hydrogen purity; (iii) controllable hydrogen production rate; (iv) adequate hydrogen storage capacity (10.8 wt.%); (v) high stability in alkaline media; and (vi) safety and pollution-free properties [4,5]. However, the borohydride hydrolysis reaction (BHR, or hydrogen evolution) in neutral and alkaline solutions is relatively slow and requires catalysts, often M^0^-nanoparticles (M^0^-NPs) [4,6,7,8,9,10,11]. The same M^0^-NPs are also used as efficient catalysts for many reductions via BH_4_^−^ [12,13,14,15,16,17,18], including environmentally essential processes [9,14,15,16,19,20,21]. Thus, catalytic reductions via BH_4_^−^ always compete with catalytic hydrolysis, and a significant excess of BH_4_^−^ is commonly required for efficient reductions [16,22,23]. An optimal reduction catalyst should be, in principle, a poor hydrolysis catalyst. Thus, the choice of the catalyst depends on the catalytic mechanisms of the reduction and hydrolysis processes.

Until recently, it was assumed that both catalytic processes start via complete borohydride hydrolysis and hydride formation on the catalyst:

BH_4_^−^ + 4H_2_O + (M^0^-NP) → B(OH)_4_^−^ + {(M^0^-NP)-H_4_}^4−^ + 4H^+^ (2)

Hydrogen formation is produced when Reaction (2) is followed by the Tafel or Heyrovsky mechanism:

(Tafel)→(M^0^-NP)-H_8_→(M^0^-NP) + 4H_2_

{(M^0^-NP)-H_4_}^4−^ + 4H^+^ (3)

(Heyrovsky)→(M^0^-NP) + 4H_2_

The reducing agents in the reduction processes are either {(M^0^-NP)-H_4_}^4−^ or (M^0^-NP)-H_8_ [24]. Alternatively, more hydrides are transformed to the M^0^-NPs, forming {(M^0^-NP)-H_n_}^n−^ that react with water to form {(M^0^-NP)-H_n+m_}^(n−m)−^, which are the source of H_2_ [24] and/or the active reducing agents [16]. This mechanism suggests that a good reduction catalyst for a given substrate is an M^0^-NP with an overpotential for H_2_ evolution higher than required for reducing the substrate.

However, it was recently shown for Ag^0^-NPs [25] and Au^0^-NPs [25] that Reaction (2) does not accurately describe the catalytic hydrolysis mechanism of BH_4_^−^ hydrolysis. The results revealed that the catalytic BHR involves fewer hydrogen atoms transferring to the M^0^-NPs than assumed. Much of the H_2_ is formed via a reaction between adsorbed H_2_O and adsorbed borohydride. The results also indicated that the mechanism depends on the nature of M and the presence of other species adsorbed to the M^0^-NPs [25]. The mechanisms of the catalytic reductions of various substrates via BH_4_^−^ on M^0^-NPs were not analyzed in detail. In principle, the following mechanisms are plausible:

(i) Reduction via adsorbed (hydrogen atoms)/hydrides formed during BHR on the surfaces of M^0^-NPs. This mechanism explains the large excess of BH_4_^−^ required for these reductions.
(ii) A direct reaction between an adsorbed BH_4_^−^ and an adsorbed substrate. This mechanism can, in principle, involve an H^.^ atom transfer, which would leave a BH_3-_ radical anion that is expected to react with water to form the BH^4−^ radical [25]. Alternatively, this reduction step might involve a hydride transfer, leaving a BH_3_ adsorbed to the surface.

Nitrophenols are common organic toxic pollutants in industrial and agricultural wastewater. 4-nitrophenol, 4-NP, is one of the refractory pollutants found in wastewater from the dye, pesticide, explosive, herbicide, and plastic industries [26]. 4-NP greatly harms the nervous system, blood cells, liver, and kidneys [26]. Therefore, converting nitrophenols to aminophenols is of significant environmental and industrial importance, particularly for aniline and paracetamol production [13,16,27]. In the presence of a catalyst, BH_4_^−^ reduces 4-NP to 4-aminophenol (4-AP) via Reaction (4), which is often employed as a model reaction to evaluate reduction catalysts [28,29,30].

4HO(C_6_H_4_)NO_2_ + 3BH_4_^−^_aq_ → 4HO(C_6_H_4_)NH_2_ + 3BO_2_^−^_aq_ + 2H_2_O (4)

Previously, the catalytic reduction of 4-NP via BH_4_^−^ in the presence of various catalysts was studied [16]. The results pointed out that the catalytic properties of different M^0^-NPs differ considerably. The order of catalytic activity reported is AgPt (9:1 alloy) > Ag > Au > AgPt (1:9 alloy) > Pt [16]. Therefore, it is of interest to measure the catalytic properties of the same M^0^-NPs as BHR catalysts with the hope that the data obtained will help design reduction catalysts that are not significantly affected by competition with the BHR catalysis. Moreover, this research will help design catalysts for hydrogen evolution without spending the BH_4_^−^ reduction power on side reactions. Notably, due to metal nanoparticle catalysts’ sustainability and stability issues, we suggest their immobilization on supports for applicative usage and to ease catalyst separation. However, it is not straightforward since even “inert” supports like silica are not innocent and change reaction mechanisms [31,32]. Due to the metal–support complex interactions, this research is beyond the scope of the current study.

## 2. Results and Discussions

### 2.1. Characterization

The complete UV-Vis characterizations of the M^0^-NPs (Ag, Au, and Pt) and BM-NPs (Ag-Pt) were reported [16,24] and are presented in Figure S1a (Supplementary Materials)**.** The absorption spectra show maxima plasmon absorptions of Ag, Au, and Ag-Pt (9:1) NPs at 396 ± 2 nm, 516 ± 2 nm, and 354 nm, respectively. The absorption spectra of the Pt^0^-NPs and Ag-Pt (1:9) BM-NPs showed only an increase in the absorption band toward the UV region. An HR-TEM image of the Ag-Pt (9:1) BM-NPs can be seen in Figure S1b; it appeared as chains of spheres attached with a particle size of 4.1 nm. HR-TEM images of M^0^-NPs are provided in Figure S2. From the HR-TEM analysis, the mean particle size diameters of the Ag, Au, Pt, and Ag-Pt NPs were 3.8, 4.4, 3.2, and 4.1 nm, respectively. The XRD pattern pointed to the alloyed crystalline structure of the BM-NPs; Figure S3 [16]. XPS analysis (Figure S4 and Table S1) showed the electronic interaction between the metals in the BM-NPs, resulting in the different catalytic activities of the studied NPs; see ESI for a detailed discussion. A complete study of the alloy structure of the Ag-Pt BM-NPs is reported elsewhere [16].

### 2.2. Sodium Borohydride Hydrolysis

#### 2.2.1. The Effect of pH

The effect of the pH on the NaBH_4_ hydrolysis was studied by adding NaBH_4_ solution to argon-saturated water; a gradual pH change was recorded, shown as the black curve in Figure S5a. A rapid increase in pH until a value of 9.0 was reached occurred due to the hydrolysis of NaBH_4_ (Reaction (1)), which slowed down until a plateau was reached at pH 9.45 due to the borate buffer formation (pKa value of boric acid is 9.24). At this point, the H_2_ evolution also slowed down—see the red curve in Figure S5b. The pH change was also recorded in the presence of 4-NP and 4-AP, in which a slower pH increase was observed above pH 8.0 in both solutions, as indicated by the red and blue curves depicted in Figure S5a. To correlate the pH with the BHR, the H_2_ production rate, upon adding NaBH_4_ to aqueous solutions at different initial pH values, was measured (Figure S5b). The curved shape of the hydrogen evolution at an initial pH of 6.9 in Figure S5b agreed with the gradual pH change shown in Figure S5a, indicating the correlation between the H_2_ evolution rate and the pH. A closer look at Figure S5a,b suggests that the rates slowed down in both curves after ca. 18 min, where the pH reached its stable value, and the H_2_ evolution became very slow (see the black curve in Figure S5a and red curve in Figure S5b of water). The hydrogen yield at this point was considerably lower than the theoretical value.

#### 2.2.2. The Competitive Effect of 4-Nitrophenol on the Catalytic BHR at Various M^0^-NP and BM-NP Catalyst Surfaces

**Figure 1:**
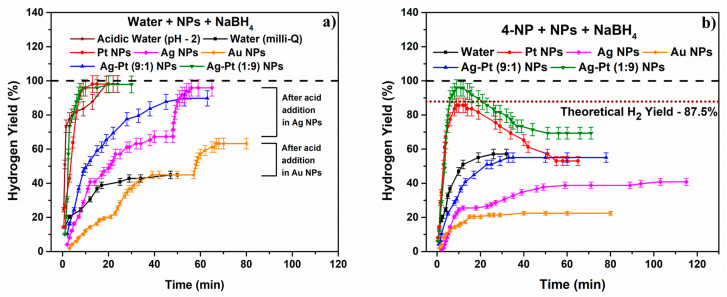
BHR kinetics on Ag, Au, and Pt M^0^-NPs and Ag-Pt (1:9) and Ag-Pt (9:1) BM-NP. (**a**) In water and (**b**) in the presence of 4-nitrophenol. The initial pH was neutral, and the pH after the reaction completion was 9.3; 11.2 µM NPs (ion-based concentration); 0.10 mM 4-NP; 0.60 mM NaBH_4_; room temperature (24 °C).

The BHR kinetics from the catalytic hydrolysis of NaBH_4_ were investigated in the presence of Ag, Au, Pt M^0^-NPs, and Ag-Pt (1:9) and Ag-Pt (9:1) BM-NPs. Figure 1a shows the BHR progress vs. time for the different catalysts; the time taken by each curve to reach a plateau is presented in Table S2 (Supplementary Materials). The BHR was very slow without a catalyst; only 45% of the hydrogen evolved within 50 min (Figure 1a black line). Almost a 100% yield of H_2_ was obtained in acidic water, with a pH value of 2.0, and Pt^0^-NPs and Ag-Pt (1:9) alloy NP catalysts; hence, these M^0^-NP and BM-NP catalysts exhibited complete and fast conversion to H_2_, relative to all studied catalysts. The Ag-Pt (9:1) BM-NP catalyst, the best catalyst for 4-nitrophenol reduction [16], showed a slow rate with a high conversion yield of 92%. Interestingly, Au^0^- and Ag^0^-NPs exhibited poor catalytic BHR: only 45 and 67% conversion at ~40 min, respectively. The gold NPs even slowed the BHR process compared to the water reaction, and the H_2_ yield at the plateau was identical to the black curve of water. After the BHR slowed to negligible rates, 13.7 mM H_2_SO_4_ was added to lower the pH to 2.0 and verify that NaBH_4_ still existed in the solution. The acid addition was followed by a rapid increase in hydrogen formation to 63 and 96% within 10 and 8 min for Au^0^- and Ag^0^-NPs, respectively (the latter results were taken from ref. [25]).

DFT analysis suggested that the final product of the Au^0^-NP-catalyzed hydrolysis was BH(OH)_2_, probably adsorbed to the Au surface [25]; however, ^11^B-NMR showed no product other than borate (Figure S6). The assumption that new non-reducing boron species were produced is corroborated by the fact that no increment in the hydrogen level was observed upon the further addition of acid to the gold NPs. To gain further insight into the reduction mechanism, the same experiments were conducted by adding 4-NP, following H_2_ formation (Figure 1b). The brown dotted line in Figure 1b denotes the theoretical expected H_2_ yield, 87.5%, considering the reducing power used to entirely reduce the 4-NP (see the calculation in the Supplementary Materials). Without a catalyst, the hydrogen formation was higher and faster in water with 4-NP compared to pure water without the 4-NP. We attribute the BHR acceleration via 4-NP to the buffer formed upon its addition. The pKa of 4-NP is 7.2 [33]; hence, it may form a buffer, where, according to Figure S5b, faster NaBH_4_ hydrolysis occurred at pH < 9. In systems containing the Pt^0^-NP or the Ag-Pt (1:9) BM-NP catalysts, a rapid H_2_ evolution was observed, as shown in Figure 1b (complete conversion with Ag-Pt (1:9) BM-NPs and 88% conversion with Pt^0^-NPs, both within 10 min).

However, after the H_2_ was formed, its volume dropped to 70 and 53% for Ag-Pt (1:9) BM-NPs and Pt^0^-NPs, respectively, within the next 40 min. This decrease in the H_2_ volume was accompanied by the disappearance of the reaction mixture’s yellow color. Thus, the drop in the H_2_ volume is attributed to the 4-NP reduction via H_2_, as already reported for Pt^0^-NPs and Ag-Pt (1:9) BM-NPs [16]. In these systems, the hydrogen cleaved into H atoms on the Pt-rich surface (it is well known that the reaction H_2_ $\overset{Pt\;NPs}{\Leftrightarrow }$ 2H_ads_ is very fast [34]), which reduced 4-NP in the next step. The H_2_ formed was still lower than the theoretical H_2_ yield (87.5%); a possible explanation for this is catalyst poisoning via 4-AP. Ag-Pt (9:1) BM-NPs, the fastest catalysts for 4-NP reduction (a complete 4-NP reduction takes place within only one min [16]), exhibited relatively slow H_2_ production, with a plateau at 55% after 33 min of reaction. Similarly, colorless solutions were observed for the Au^0^ and Ag^0^ NPs after 8 and 5 min, whereas only 22% and 41% hydrogen yields were obtained after 40 and 103 min, respectively. Table 1 shows the observed rate constant (*k*_obs_) values for BHR from the catalytic NaBH_4_ hydrolysis on M^0^-NPs and BMNPs in the absence and presence of 4-NP, as derived from Figure 1.

Without 4-NP, Ag-Pt (1:9) NPs showed the highest *k*_obs_ value of 8.2 × 10^−3^ s^−1^ compared to the other catalysts. The *k*_obs_ values for all NPs increased in the presence of 4-NP except for Ag-Pt (1:9) NPs, which decreased almost by half. This minor acceleration of the BHR possibly stemmed from the relative adsorption of borate and 4-NP on the various catalysts. The 4-NP maintained a lower pH—Figure S5a—which may have accelerated the BHR. The reverse trend of Ag-Pt (1:9) NPs suggests a different affinity of the catalyst surface and, therefore, different catalytic activity. UV-visible spectra of the product suspensions confirmed the formation of 4-AP at 300 nm at a concentration equal to the 4-NP introduced (Figure S7), hence the full conversion of 4-NP to 4-AP. Since all the NPs synthesized had spherical morphologies in the size range of 3–5 nm and were produced without any added stabilizer, we attributed the catalytic performance differences between the catalysts to the nature of the metal and alloy composition.

**Figure 2:**
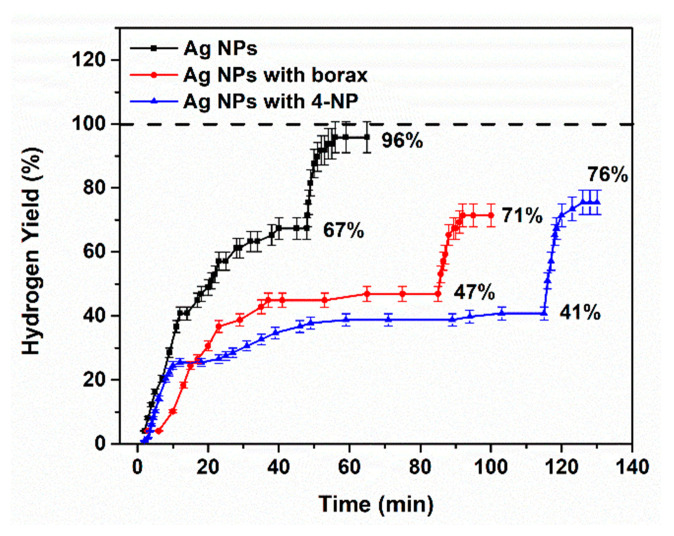
BHR kinetics on the surface of Ag^0^-NPs. In water (black), borax (red), and 4-NP (blue). H_2_SO_4_ was added in each experiment after reaching the plateau. Initial pH 9.16 (borax reaction), 6.9 (other reactions); 0.60 mM borax; 11.2 µM Ag^0^-NPs; 0.10 mM 4-NP; 0.60 mM NaBH_4_; 13.7 mM H_2_SO_4_; room temperature (24 °C).

No full hydrogen yield was obtained for Ag^0^-NPs; the H_2_ evolution nearly ended at 67% yield but increased to 96% after adding acid (Figure 1a pink curve). The low H_2_ formation before acid addition may have been due to catalyst poisoning by the borate product (which probably desorbed after its protonation by adding acid) or surface poisoning by 4-AP when 4-NP was added. Therefore, to check the poisoning effect of borate on Ag^0^-NPs, borax that reacts with water to yield borate (0.60 mM, pH 9.16) was added to the Ag^0^-NPs before performing the reaction, and the hydrogen evolution was monitored; Figure 2. This figure also shows the BHR on the surface of Ag^0^-NPs in the presence of 4-NP. In all cases, 13.7 mM H_2_SO_4_ was added at the end of the reaction. The results indicated that borate, the hydrolysis product of NaBH_4_, slowed down the BHR rate and decreased the evolved hydrogen by ca. 30%. Adding acid at the end of the hydrogen evolution elevated the hydrogen level from 47% to 71% without reaching 100% H_2_ yield. This confirmed that the catalyst’s surface was poisoned by borate, which prevented the catalytic hydrolysis of borohydride on the Ag^0^-NP surface.

Moreover, the pKa of the adsorbed borate might have differed from that in the solution. Another possibility is that plausibly, some of the metal surfaces, e.g., Au^0^ and Ag^0^, catalyzed the polymerization of B(OH)_4_^−^, B(OH)_3_, BH_n_(OH)_4−n_^−^, and BH_n_(OH)_3−n_ that coated the metal surface and possibly inhibited the contact of H_3_O^+^ with the encapsulated B-H bonds. When 4-NP was added to the Ag^0^-NPs, some of the reducing power went to reduce the 4-NP; Figure 1b. The acid addition elevated the hydrogen level from 41% to 76%; however, this was still lower than the theoretical value of 87.5%.

#### 2.2.3. The Effect of 4-Nitrophenol Concentration

**Figure 3:**
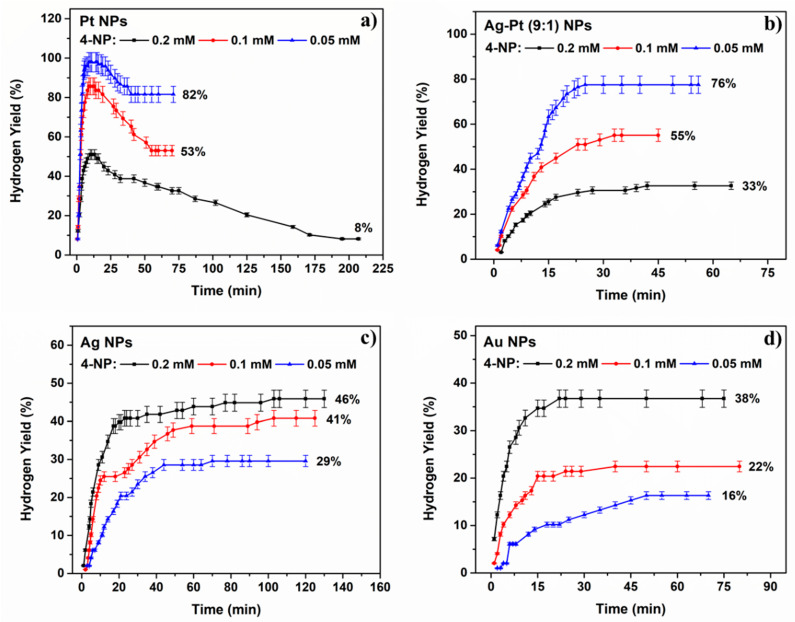
NaBH_4_ hydrolysis in the presence of various concentrations of 4-NP. A constant concentration of catalyst (11.2 µM) and NaBH_4_ (0.60 mM) on the surface of (**a**) Pt, (**b**) Ag-Pt (9:1), (**c**) Ag, and (**d**) Au NPs. The experiments were performed at 24 °C. Complete transformations of 4-NP to 4-AP were achieved during these experiments.

The effect of the 4-NP concentration on the kinetics of the catalytic hydrogen evolution due to NaBH_4_ hydrolysis was explored. Constant concentrations of NaBH_4_ (0.60 mM) and NPs (11.2 µM) were used; the 4-NP concentrations were 0.20, 0.10, and 0.050 mM. Figure 3 presents the results of these experiments. The Pt^0^-NPs and Ag-Pt (9:1) BM-NPs followed a consistent trend in which the amount of hydrogen evolved decreased when the 4-NP concentration increased, suggesting that beyond the reducing power that went for 4-NP reduction, the 4-AP product affected the H_2_ evolution (the H_2_ was lower in the time region in which the 4-NP was already completely transformed into 4-AP—60 min and 1 min for Pt^0^-NPs and Ag-Pt (9:1) BM-NPs, respectively). In contrast, Ag^0^-NPs and Au^0^-NPs followed a reverse trend; the amount of hydrogen evolved increased with the 4-NP concentration. This opposite trend might be attributed to inhibiting the borate polymerization via the adsorbed product, 4-AP. Therefore, the surface effects of borate and 4-AP had a significant role in the competitive system studied and affected the kinetics and yields.

#### 2.2.4. The Effect of the 4-Aminophenol Product

**Figure 4:**
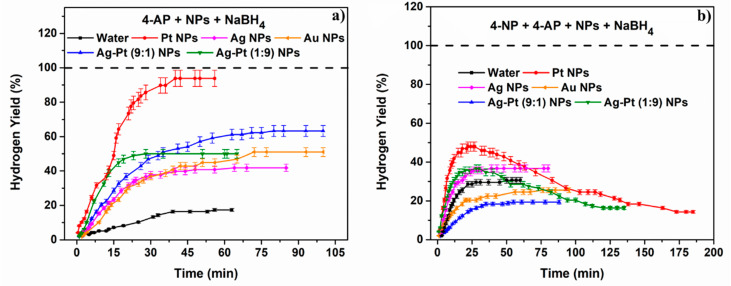
Effect of 4-AP on the rate and yield of H_2_ via the catalytic BHR. (**a**) In the presence of 4-AP, and (**b**) in the presence of 4-NP and 4-AP at room temperature (24 °C). Catalyst concentration: 11.2 µM; [4-NP] = 0.10 mM; [NaBH_4_] = 0.60 mM; [4-AP] = 0.10 mM.

Like the 4-NP effect on the BHR, its product, 4-AP, also affected the rate and yield of H_2_ evolution in the investigated system. The effect of 4-AP on the BHR was studied, as demonstrated in Figure 4. In addition, Table S2 sums up the H_2_ percentage of each catalyst at the plateau at the end of the experiment and the time required to reach it. A comparison between the results in Figure 1a and Figure 4a indicates that adding 4-AP significantly slowed the hydrogen formation rate and decreased its yield on all the catalysts. 4-AP also slowed the BHR without a catalyst, forming only 17.3% of H_2_, less than half the yield in the absence of 4-AP; Figure 1a. Pt^0^-NPs exhibited 94% hydrogen evolution in the presence of 4-AP only after 40 min, much slower than in its absence; Figure 1a. In the presence of 4-AP, Ag-Pt (1:9) BM-NPs exhibited a remarkable decrease in the hydrogen yield, forming only 50% of hydrogen within 28 min, almost half of the H_2_ yield in the absence of 4-AP. Interestingly, the hydrogen yield in the presence of Au^0^-NPs was not affected by the presence of 4-AP, but the evolution rate was slower. As Figure 4b shows, when 4-NP was added, the most prominent effect was observed for the Pt^0^-NPs, Ag-Pt (1:9), and Ag-Pt (9:1) BM-NPs, and a smaller effect was observed for Au^0^-NPs (see also Table S2). However, for Ag^0^-NPs, no significant effect was observed. Compared to Figure 4a, adding 4-NP accelerated the BHR without a catalyst, probably by maintaining a low pH in the solution (Figure S5a).

Hence, the effect of 4-AP on the BHR may be due to several reasons: (1) 4-AP was probably adsorbed on some of the catalyst surfaces and poisoned them; (2) the pKa of 4-AP is 5.43 [35]; therefore, it was present in its alkaline form, which might have affected the BHR, for example, via Reaction (5).

BH_3_OH^−^ + H_2_NC_6_H_4_OH ⇌ H_3_BNH_2_C_6_H_4_OH + OH^−^ (5)

If the latter equilibrium is shifted to the right, it will raise the pH. Furthermore, H_3_BNH_2_C_6_H_4_OH probably hydrolyzes slower than BH_3_OH^−^. It is well known that different M^0^-NPs catalyze the hydrolysis of H_3_BNH_2_ differently [36,37,38,39,40]. Yao et al. studied the catalytic hydroboration reduction of amides to amines and focused on such products [41,42,43]. A detailed investigation of the 4-AP effect on the BHR rate and yield is beyond the scope of this study.

## 3. Materials and Methods

### 3.1. Materials

All chemicals were of analytical grade and used without further purification. Potassium hexachloroplatinate (K_2_PtCl_6_), silver sulfate (Ag_2_SO_4_), tetra-chloro-auric acid (HAuCl_4_), and sodium borohydride (NaBH_4_) were purchased from Strem Chemicals (Newburyport, MA, USA). 4-NP and 4-AP were purchased from Sigma (Rehovot, Israel). Sodium hydroxide (NaOH) and sulfuric acid (H_2_SO_4_) were purchased from Alfa Aesar (Ward Hill, MA, USA) and Merck (Boston, MA, USA). Millipore water of a resistivity > 15 MΩ·cm was used throughout the experiments.

### 3.2. Methods and Instrumentation

To follow the reaction kinetics, we used a manometer-based set-up. The reaction was performed in a 50 mL two-neck round bottom flask reactor; a U-tube manometer was connected to one of the necks with a pipe; the solution was purged with Ar through a rubber septum on the other neck that was also used for reagent additions. The water level in the arms of the manometer was balanced before the last reagent (NaBH_4_) was added. The BHR kinetics was followed by reading the water level in the U-tube manometer. The hydrogen pressure was calculated according to Equation S1 in the Supplementary Materials.

The ultraviolet–visible (UV-Vis) absorption spectra were measured using an Agilent 8453 UV-visible spectrophotometer (Agilent Technologies. Inc., Santa Clara, CA, USA). An inductively coupled plasma–optical emission spectrometer (ICP-OES, Ametek, SPECTRO ARCOS, Kleve, Germany) was used to determine ionic concentrations. High-resolution transmission electron microscopy (HR-TEM) analyses were obtained with a Tecnai 12 microscope (Thermo Fisher Scientific, Hillsboro, OR, USA). The X-ray diffraction (XRD) patterns were recorded in the 2θ range of 20°–80° with Cu Kα radiation (λ = 0.154 nm) and a step size of 0.02° and 5 s per step to study the structural analysis and phase composition of Ag−Pt alloy NPs using a Panalytical X’Pert Pro X-ray powder diffractometer (Malvern Panalytical Ltd., Malvern, United Kingdom). The oxidation states and the binding energies of the NPs were studied via X-ray photoelectron spectroscopy (XPS) using an (XPS/AES) ESCALAB 250 (Thermo Fisher Scientific, Waltham, MA, USA). The samples were drop-dried onto a silicon substrate. The ^11^B NMR spectra were recorded on a 400 MHz Bruker Avance III NMR spectrometer (Bruker Corporation, Billerica, MA, USA) calibrated using BF_3_·Et_2_O in CDCl_3_ (0.0 ppm) as a standard reference sample in a boron-free quartz tube. The spectra were obtained using a zgbs pulse sequence with LB 100 Hz experimental line broadening.

### 3.3. Catalyst Preparation

The M^0^-NP and M/M’-NP bimetallic alloy NPs, BM-NP, suspensions were prepared without the addition of any stabilizer, as reported elsewhere, using the modified Creighton’s procedure [44] by Zidki et al. [16,45]. Briefly, 30 mL of 2.0 mM ice-cold NaBH_4_ aqueous solution was added at once under vigorous stirring to 10 mL of 1.0 mM of the desired precursor salts dissolved in water (HAuCl_4_, and K_2_PtCl_6_ for Au, and Pt M^0^-NPs, and 0.5 mM Ag_2_SO_4_ for Ag M^0^-NPs, respectively, or a mixture at the specific molar ratio of these salts in the case of BM-NPs). The final metal ion concentration was 2.5 × 10*^−^*^4^ M. All the NP concentrations are stated as ion-based M^n+^ concentrations in all the experiments. The suspensions were ultrafiltrated using an Amicon Stirred Cell Model 8050 (Merck Millipore, Burlington, MA, USA) equipped with a Hydrosart^®^ membrane of 5K MWCO by Sartorius to verify that all the ions were reduced. The filtrate was analyzed via ICP-OES (Ametek, SPECTRO ARCOS, Kleve, Germany). The metal ion concentrations were below the detection limit; hence, a full reduction of the metal precursors was achieved. The resulting suspensions were yellow and ruby red for Ag and Au M^0^-NPs, respectively, and black for the Pt^0^-NPs and Ag-Pt BM-NPs. The pH of the M^0^- and BM-NPs was ~9.0 due to the borate buffer formed during the NP synthesis and the borohydride hydrolysis. Note that all the M^0^-NPs had some oxides on their surfaces, which were partially responsible for maintaining their stability in aqueous suspensions.

### 3.4. Hydrogen Evolution Experiments

**Scheme 1:**
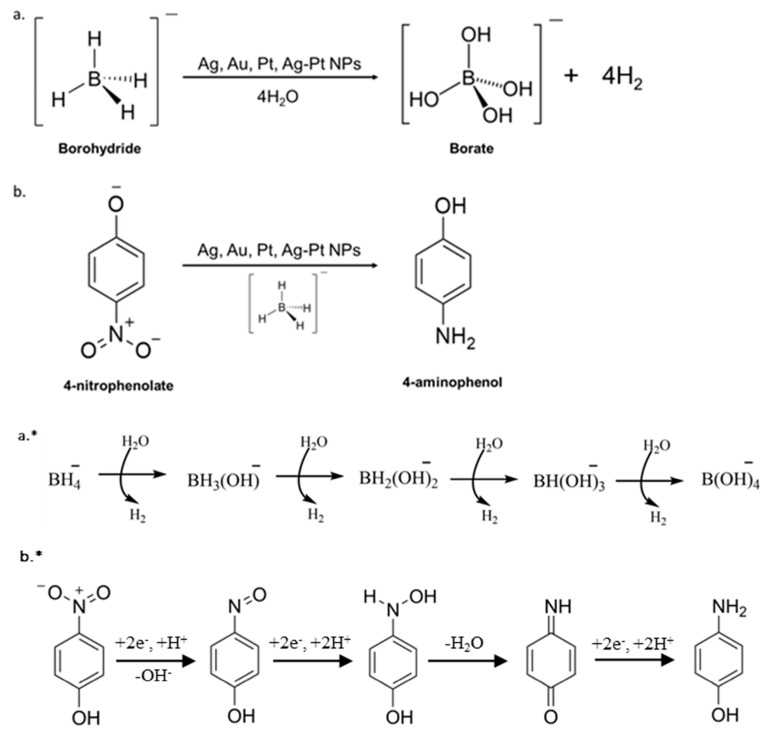
The possible borohydride reactions on various catalysts. (**a**) Borohydride hydrolysis resulting in H_2_, and (**b**) reduction of 4-NP to 4-AP via borohydride on the surface of NPs. Under our working conditions (pH~9), the borate occurs both as B(OH)_3_ and B(OH)_4_^−^ and 4-NP as to 4-nitrophenolate. (a*) Reaction channel of borohydride hydrolysis on Au^0^-NPs. (b*) Reaction steps for the reduction of 4-nitrophenol to 4-aminophenol (adapted with permission from Ref. [25], © 2023 John Wiley & Sons, Inc.).

In a typical procedure, 50 mL of the desired solution (water, 4-NP, or 4-AP) was mixed with 2.35 mL of M^0^-NPs or BM-NPs (to give 11.2 µM as the metal ion concentration) in the reactor and purged with Ar for 15 min under stirring. The water level of the manometer arms was balanced before the last added reagent. Then, 0.30 mL of Ar-purged freshly prepared 0.106 M NaBH_4_ solution (a final concentration of 6.0 × 10*^−^*^4^ M in the reactor) was quickly added to the reactor to initiate the catalytic reaction. All the reactions were performed at room temperature (24 °C). A concentrated NaBH_4_ solution was used to minimize its volume so that the water level in the manometer did not change appreciably. As the reaction proceeded, the water level in the manometer was recorded at different times. The readings provided the hydrogen evolution rate for each catalyst in the absence or presence of 4-NP. In the presence of 4-NP, as the reaction proceeded, the yellow color of the reaction mixture (due to 4-nitrophenolate absorption) gradually disappeared due to the formation of 4-AP, depending on the catalytic activity of the M^0^- and BM-NPs [16]. The resulting data were plotted as hydrogen yield vs. time. Theoretically, the complete conversion of NaBH_4_ to hydrogen (100% H_2_ yield) would give 126.36 µmol of H_2_ and 479 Pa gauge pressure (calculated using the ideal gas equation; see the Supplementary Materials). This value is denoted in the figures by a dashed line. Each data point of hydrogen yield is the average of three independent measurements; the graphs have an experimental error limit value of ±5%. NaOH and H_2_SO_4_ were used to adjust the initial pHs to study the pH effect on the BHR kinetics. Scheme 1 represents the catalytic hydrolysis of borohydride (a) and the catalytic reduction of 4-NP to 4-AP via borohydride (b) on the surface of Ag, Au, Pt M^0^-NPs, and Ag-Pt BM-NPs. Scheme 1a* suggests a sequential four-step intermediate reaction mechanism for catalyzed borohydride hydrolysis on Au^0^-NPs [25], and Scheme 1b* shows the general intermediate reaction mechanism for the conversion of 4-NP to 4-AP [46,47].

## 4. Conclusions

Ag, Au, Pt M^0^-NPs, and Ag-Pt (9:1), Ag-Pt (1:9) BM-NPs were studied as catalysts for the hydrogen evolution from NaBH_4_. M^0^-NPs catalyze both the BHR and the 4-NP reduction; hence, the competition between the two reduction processes is of interest. The observation that the reduction of 4-NP by BH_4_^−^ catalyzed by Pt^0^-NPs followed the reaction sequence of BH_4_^−^ + H_2_O → H_2_ + B(OH)_4_^−^, followed by H_2_^−^ + 4-NP → 4-AP points out that H atoms adsorbed on Pt^0^-NPs reduced 4-NP. However, the observation that the *k*(red)_obs_ for the reduction of 4-NP on Ag^0^, Ag-Pt (9:1), and Au^0^ were considerably higher than *k*(BHR)_obs_ for the same catalyst indicated that most of the reductions do not occur via hydrogen atoms adsorbed on the surface of the catalysts. It should be noted that both processes depend on several factors: the surface of the catalyst and its electron affinity, the species adsorbed to the surface of the catalyst, the solution pH, etc. Thus, the detailed mechanism for each reaction on each catalyst has to be separately evaluated, as was performed recently for the BHR process on Ag^0^ [25] and Au^0^ [25].

## Figures and Tables

**Table 1:** Observed rate constants for catalytic BHR and catalytic 4-NP reduction.

	Absence of 4-NP ^a^	Presence of 4-NP ^b^	4-NP-Red ^c^
Sample	*k*(BHR)_obs_ × 10^−3^ s^−1^	*k*(BHR)_obs_ × 10^−3^ s^−1^	*k*(Red)_obs_ × 10^−3^ s^−1^
Pt	3.8	5.9	0.8
Ag-Pt (1:9)	8.2	4.5	2.7
Ag-Pt (9:1)	1.4	1.6	59
Ag	1.1	1.2	13.2
Au	0.46	2.2	4.9
Water	1.3	2.4	---

^a^ Catalyst concentration: 11.2 µM; [NaBH_4_] = 0.60 mM, pH~9. ^b^ Catalyst concentration: 11.2 µM; [4-NP] = 0.10 mM; [NaBH_4_] = 0.60 mM, pH~9. ^c^ Data were taken from ref. [16] Catalyst concentration: 12.0 µM; [NaBH_4_] = 0.635 mM, [4-NP] = 0.102 mM; pH~9.

## Data Availability

The data presented in this study are available in the article and the Supplementary Materials.

## Supplementary Materials

The following supporting information can be downloaded at: https://www.mdpi.com/article/10.3390/molecules28186530/s1, Figure S1: UV-Vis spectra of NPs and HR-TEM image of Ag-Pt (9:1) BM-NP; Figure S2: HR-TEM images of Ag0-NPs, Au0-NPs, and Pt0-NPs; Figure S3: XRD pattern of Ag-Pt (9:1) NPs; Figure S4: Regional XPS spectra of various NPs; Table S1: Binding energies for Ag, Pt, and Ag-Pt alloy NPs from Ag3d and Pt4f XPS spectra; Figure S5: pH changes vs. time of NaBH4 hydrolysis with water, 4-nitrophenol, and 4-aminophenol, and hydrogen evolution kinetics of water solutions with different initial pHs; Table S2: Hydrogen yields from the catalytic NaBH4 hydrolysis on nanoparticles in the absence and presence of 4-NP and 4-AP; Figure S6: 11B NMR spectra of Au NP-catalyzed NaBH4 hydrolysis in H2O/D2O (90:10) solvent under Ar; Figure S7: UV-Vis spectroscopy scan after the reaction was completed with 4-NP.

## Sample Availability

Sample information is available from the authors.
