# Supplementary material for: The Competition between 4-Nitrophenol Reduction and BH4− Hydrolysis on Metal Nanoparticle Catalysts
Source: Molecules. 2023 Sep 8;28(18):6530. doi: 10.3390/molecules28186530 (PMC10535303; doi:10.3390/molecules28186530)
Supplement: Supplementary file 1 [file molecules-28-06530-s001.zip › molecules-2562647-supplementary.pdf]

Supplementary Materials

The Competition between 4-Nitrophenol Reduction and BH_4^- Hydrolysis on Metal Nanoparticle Catalysts

Shalaka Varshney[1], Dan Meyerstein[1,2], Ronen Bar-Ziv[3], and Tomer Zidki[1,]*

[1] Chemical Sciences Department, The Centers for Radical Reactions and Material Research, Ariel University, Kyriat Hamada 3, Ariel 40700, Israel.

[2] Department of Chemistry, Ben-Gurion University of the Negev, Beer-Sheva 84105, Israel.

[3] Department of Chemistry, Nuclear Research Centre Negev, Beer-Sheva 84190, Israel.

Corresponding Author

* Email: tomerzi@ariel.ac.il

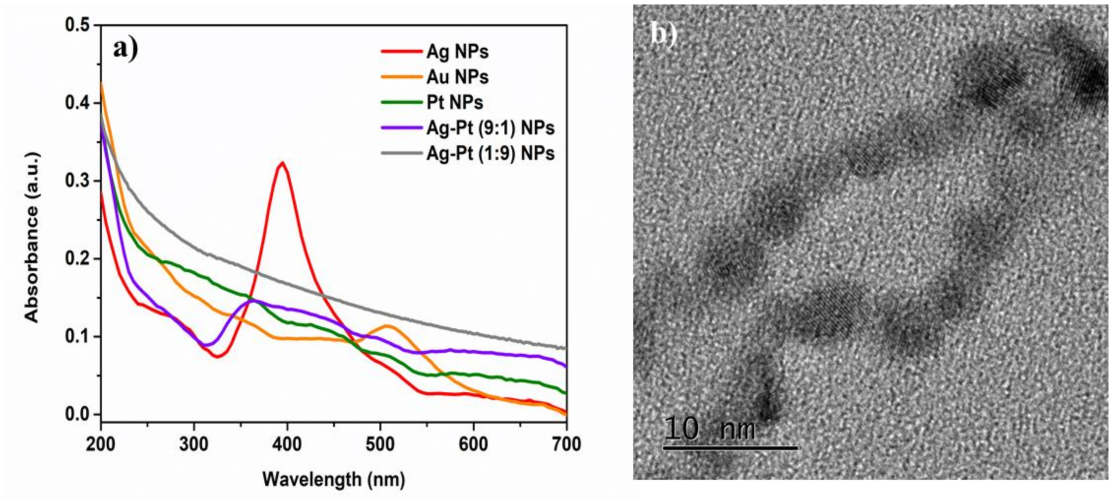

Figure S1. a) UV-Vis spectra of Ag, Au, Pt M^0-NPs and Ag-Pt (9:1), Ag-Pt (1:9) BM-NPs. b) HR-TEM image of Ag-Pt (9:1) BM-NP.

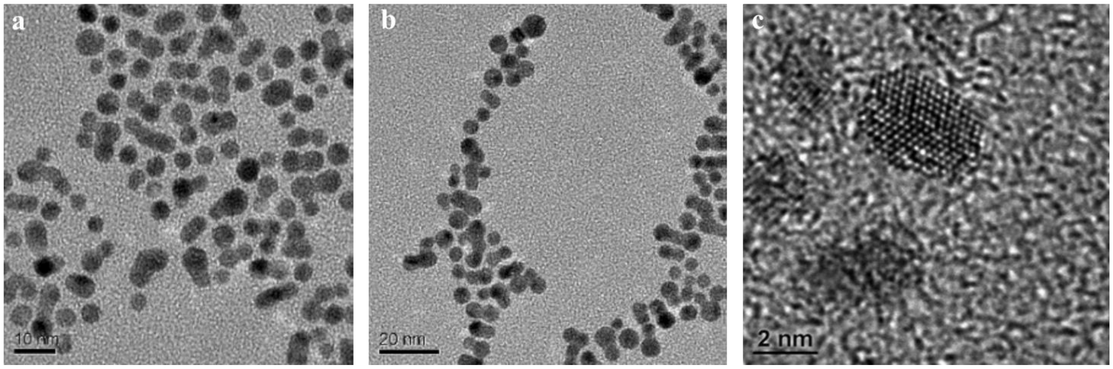

Figure S2. HR-TEM images of a) Ag^0-NPs, b) Au^0-NPs, and c) Pt^0-NPs.

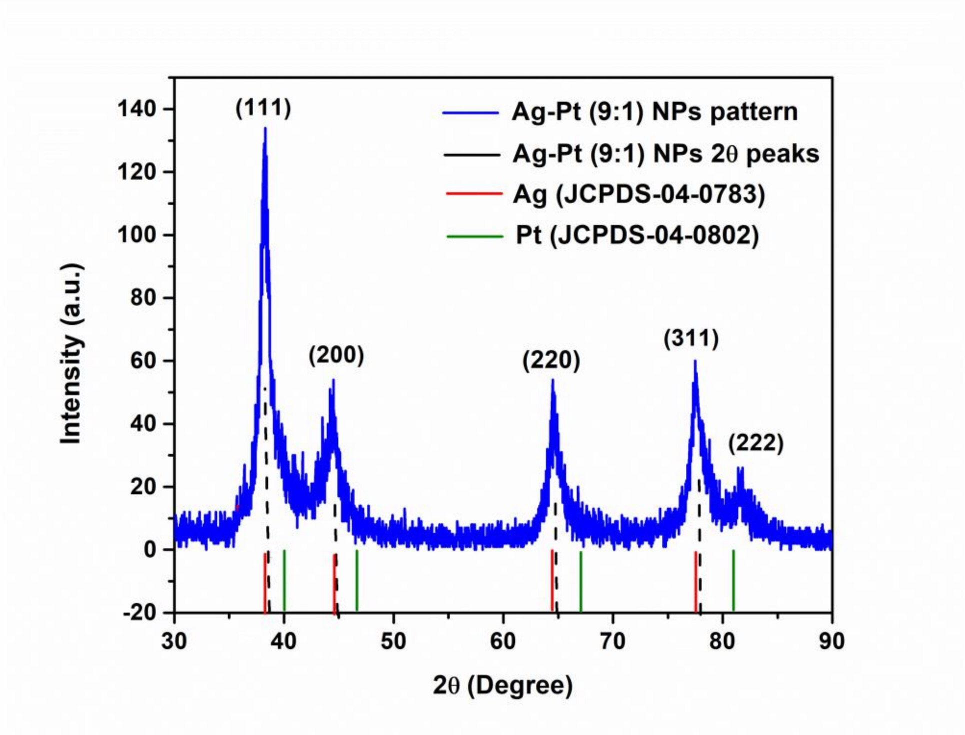

Figure S3. XRD pattern of Ag-Pt (9:1) NPs. The pattern peaks' dashed lines lie between the lines of the Ag (red) and Pt planes (green).

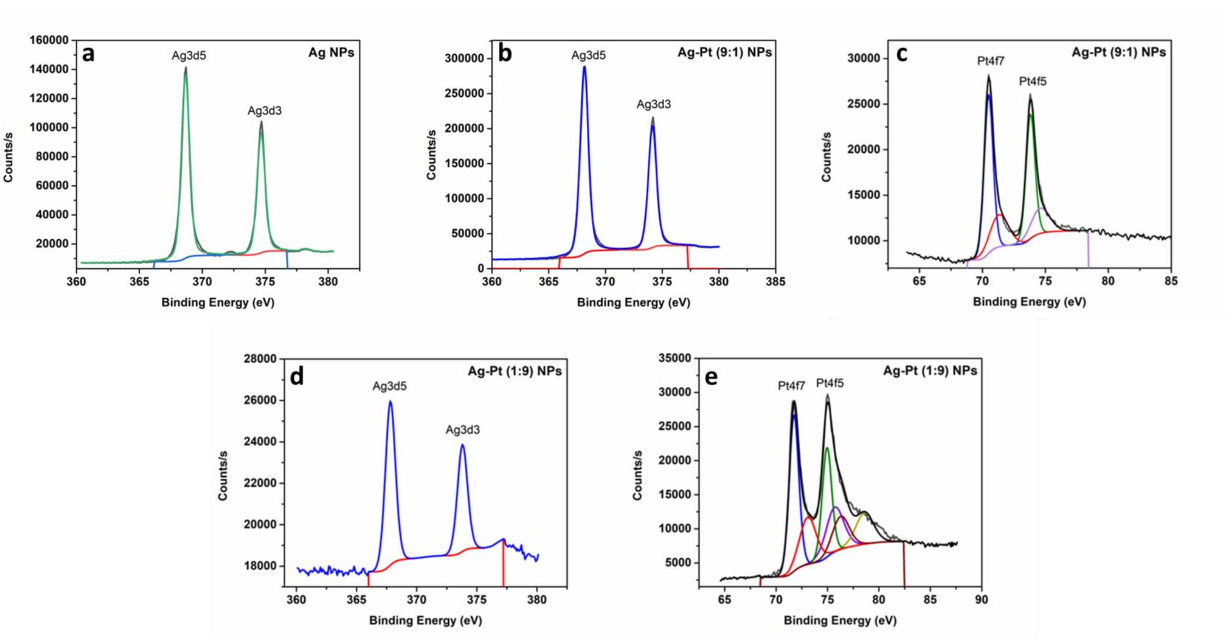

Figure S4. Regional XPS spectra of various NPs: a) Ag3d of Ag^0-NPs, b) Ag3d of Ag-Pt (9:1) NPs, c) Pt4f of Ag-Pt (9:1) alloy NPs, d) Ag3d of Ag-Pt (1:9) NPs, and e) Pt4f of Ag-Pt (1:9) NPs. Note: The results in Figure S4a,b,c are adapted from reference [1].

The X-ray photoelectron spectroscopy (XPS) analysis in Fig. S4 demonstrates the regional Ag3d and Pt4f spectra of the metal and alloy NPs. The Ag3d and Pt4f binding energies are tabulated in Table S1. The regional spectrum of the Pt4f in Ag-Pt (9:1) NPs shows a doublet with binding energies at 70.5 and 73.8 eV, indicating the existence of the Pt metallic state. In comparison with pure Pt (71.8 eV, 75.1 eV), the Ag-Pt (9:1) NP binding energies of Pt downshifts, demonstrating an electron transfer from the less electronegative Ag (1.93) to Pt with a higher electronegativity (2.28). The binding energies of metallic Ag are located at 368.1 and 374.1 eV, whereas the binding energies of pure Ag are at 368.6 and 374.6 eV. Ag and Pt binding energies shifted from their energies of the pure metals (Table S1), indicating alloy formation and that their surface electronic structure has changed upon different alloy compositions. These binding energy shifts affect the NP activities and selectivities due to the ensemble nature, and the ligand effects arise.

Table S1. Binding energies for Ag, Pt, and Ag-Pt alloy NPs from Ag3d and Pt4f XPS spectra.

**Sample**	**Metal**	**Binding energy (eV)**
**Ag^0-MPs**	Ag	368.6. 374.6
**Pt^0-NPs**	Pt	71.8, 75.1
**Ag-Pt (9:1) NPs**	Ag	368.1, 374.1
	Pt	70.5, 73.8
**Ag-Pt (1:9) NPs**	Ag	368.8, 373.8
	Pt	71.8, 75.0

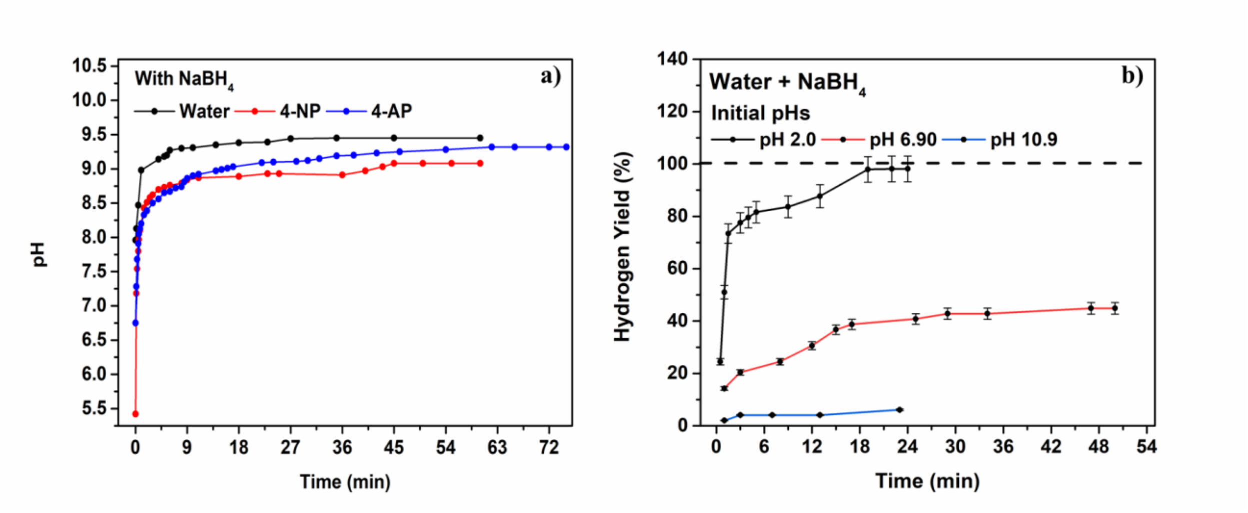

Figure S5. a) pH changes vs. time of $NaBH_4$ hydrolysis with water, 4-nitrophenol, and 4-aminophenol, and b) hydrogen evolution kinetics of water (pH 6.9), solutions with initial pH of 2.0 and 10.9. The experiments were conducted at room temperature (24 ºC), $[NaBH_4]$ = 0.60 mM.

Figure S5b shows a rapid H_2 evolution at an initial pH of 2.0, slower in water (pH 6.9), and extremely slow at an initial pH of 10.9. It is clear that borohydride hydrolysis above pH 9 is very slow at room temperature, as shown in Figure S5. The pH increases due to the formation of the basic metaborate ion. Upon the addition of NaOH to water, the $NaBH_4$ hydrolysis process ceases [2]. Therefore, the hydrogen generation process becomes considerably slower in alkaline media, whereas borohydride hydrolysis is rapidly completed at a low pH. The H_2 yield obtained at low pH agrees with the calculated amount of H_2 from the $NaBH_4$ concentration, within the experimental error (see below and the dashed line, Figure S5b).

Calculating the pressure of the formed hydrogen:

$$P(H_2) = P(atm) + \rho g h, \quad (1)$$

where $P(H_2)$ is the pressure of hydrogen in Pa; P(atm) is the atmospheric pressure (101,325 Pa), ρ is the water density (997 $kg \cdot m^{-3}$), g is the gravitational acceleration (9.8 $m \cdot s^{-2}$), and h is the height increment of the water.

The hydrogen yield percentage of the stoichiometric amount of H_2 was calculated from the pressure that hydrogen produced, as follows:

Pressure of H_2

$P(H_2) = \rho gh + P_{atm}$

Height at full H_2 yield = 4.81 cm

$P(H_2) = 997\ kg/m^3 \times 9.8\ m/s^2 \times (4.81\times 10^{-2})\ m + 101{,}325\ Pa$

$P(H_2)$ = 101,795 Pa (Total pressure of H_2 obtained in manometer)

Gauge Pressure: 101,795-101,325 = 470 Pa

Theoretical amount of H_2 from $NaBH_4$ hydrolysis:

The solution volume is 52.65 mL.

$[NaBH_4]$ = 0.60 mM

0.6 mM × 4 = 2.4 mM of H^-

2.4 mM × 52.65 mL = 126.36 μmol of H_2

Taking the amount of H_2 from $NaBH_4$ and the total pressure obtained, we cross-checked the volume of the gas obtained in the manometer.

The total height increment of water in the manometer due to H_2 evolution is 4.81 cm, which corresponds to a volume of 3.01 mL.

Through the ideal gas equation:

$$PV = nRT, \tag{2}$$

where P is the pressure of H_2 in atm, V is the volume of H_2 in liters, n is the amount of H_2 in moles, R is the ideal gas constant (0.0821 L·atm/(mol·K), and T is the absolute temperature in Kelvin (24ºC; 297.15 K)

The total pressure of H_2 obtained is 101,795 Pa.

The total amount of H_2 from $NaBH_4$ is 126.36 μmol.

Setting the values in (2):

$(101{,}795/101{,}325)\ atm \times V\ (L) = 126.36 \times 10^{-6}\ M \times 0.0821\ L\cdot atm/(mol\cdot K) \times 297.15\ K$

V = 3.07 mL

Therefore, the mass of borohydride added, and the resulting pressure and volume of hydrogen obtained are correlated.

Theoretical yield of hydrogen after 4-NP reduction

$4\text{-NP} + 6\ H^+ + 6\ e^- \rightarrow 4\text{-AP} + 2H_2O$

[4-NP] = 0.100 mM

Each 4-NP takes 6 electrons for its reduction to 4-AP.

$0.100\ mM \times 6 = 0.60\ e^-_{4\text{-NP}}$

$[NaBH_4]$ = 0.60 mM

Each $NaBH_4$ has 8 electrons.

$0.60\ mM \times 8 = 4.8\ e^-_{NaBH4}$

$[e^-_{NaBH4}] - [e^-_{4\text{-NP}}]$ = Theoretical concentration of the available electrons after 4-NP reduction

4.8 – 0.60 = 4.2

(4.2/4.8) × 100 = 87.5% (theoretical H_2 yield in the presence of 4-NP).

12.5% is needed for 4-NP reduction.

Table S2. Hydrogen yields from the catalytic $NaBH_4$ hydrolysis on nanoparticles in the absence and presence of 4-NP and 4-AP.*

Sample	Hydrogen Yield (%)			
	**Absence of 4-NP and 4-AP**	**Presence of 4-NP**	**Presence of 4-AP**	**Presence of 4-NP and 4-AP**
**Water**	45 (50)	57 (32)	17 (56)	31 (50)

**Acidic Water**	98 (22)			
**Pt**	98 (13)	53 (55)	94 (40)	14 (173)
**Ag-Pt (1:9)**	98 (20)	70 (51)	50 (28)	16 (120)
**Ag-Pt (9:1)**	92 (52)	55 (33)	63 (80)	19 (55)
**Ag**	67 (40)	41 (103)	42 (64)	37 (39)
**Ag after acid addition****	96 (8)			
**Au**	45 (39)	22 (40)	51 (72)	26 (76)
**Au after acid addition****	63 (10)			

Catalyst concentration: 11.2 μM; [4-NP] = 0.100 mM; $[NaBH_4]$ = 0.60 mM; [4-AP] = 0.100 mM
*The values in parentheses denote the time for reaction completion in minutes.
** H_2SO_4 was added after reaching a plateau to reduce the pH to 2.0.

Fig. S6 shows the ^{11}B NMR spectra recorded after H_2 formation in the Au NP-catalyzed hydrolysis of $NaBH_4$ under Argon. The spectra were recorded for the entire chemical shift range of the boron NMR to check whether any non-reducing boron species is formed that restricts the H_2 formation. It is clear that ^{11}B NMR showed no product other than borate (1.93 ppm) (Fig. S6).

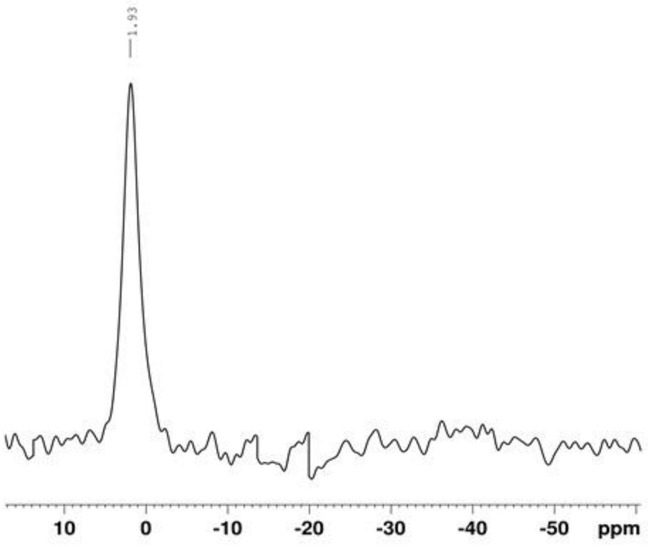

Figure S6. ^{11}B NMR spectra of Au NP-catalyzed $NaBH_4$ hydrolysis in H_2O/D_2O (90:10) solvent under Ar.

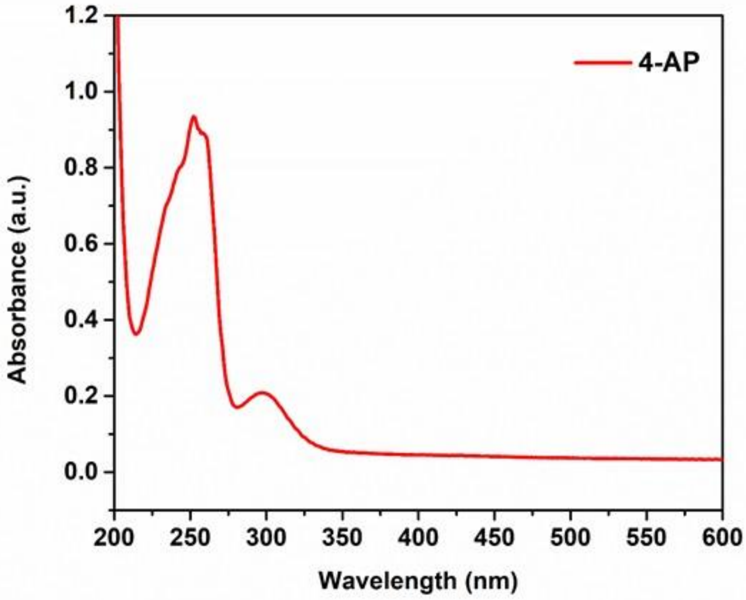

Figure S7. UV-Vis spectroscopy scan after the reaction was completed with 4-NP.

The UV-vis spectrum shows the formation of 4-AP at a wavelength of 300 nm. The calculated molar absorption coefficient of 4-AP at 300 nm is 1,900 $L\cdot mol^{-1}\cdot cm^{-1}$. The absorbance at 300 nm in Fig. S7 is 0.2.

According to the Lambert-Beer Law:

$A = \varepsilon\cdot l\cdot c$

$C = 0.200/1{,}900 = 1.00 \times 10^{-4}$ M

This concentration of 4-AP matches the starting concentration of 4-NP, i.e., 0.100 mM.

References

1. Varshney, S.; Bar-Ziv, R.; Zidki, T. On the Remarkable Performance of Silver-based Alloy Nanoparticles in 4-nitrophenol Catalytic Reduction. *ChemCatChem* **2020**, *12*, 4680–4688. https://doi.org/10.1002/cctc.202000584.
2. Schlesinger, H.I.; Brown, H.C.; Finholt, A.E.; Gilbreath, J.R.; Hoekstra, H.R.; Hyde, E.K. Sodium Borohydride, Its Hydrolysis and Its Use as a Reducing Agent and in the Generation of Hydrogen. *J. Am. Chem. Soc.* **1953**, *75*, 215–219. https://doi.org/10.1021/ja01097a057.
